# Prevalence and Associated Factors of Normal-Weight Central Obesity Among Community-Dwelling Persons 45 Years and Older in Indonesia in 2023

**DOI:** 10.1007/s10823-026-09575-y

**Published:** 2026-04-25

**Authors:** Karl Peltzer, Supa Pengpid

**Affiliations:** 1 Department of Psychology, University of the Free State, Bloemfontein, Republic of South Africa; 2 Department of Health Education and Behavioral Science, Faculty of Public Health, Mahidol University, Bangkok, Thailand; 3 Department of Medical Research, China Medical University Hospital, China Medical University, Taichung, Taiwan; 4 Department of Psychology, College of Medical and Health Science, Asia University, Taichung, Taiwan; 5 Department of Education and Behavioral Science, Faculty of Public Health, Mahidol University, Bangkok, Thailand; 6 Department of Public Health, Sefako Makgatho Health Sciences University, Pretoria, Republic of South Africa (RSA)

**Keywords:** Normal weight central obesity, Adults 45 years and older, Community survey, Indonesia

## Abstract

Our study sought to investigate the prevalence and related factors of normal-weight central obesity (NWCO) among people aged 45 and over, using national community-dwelling data from the 2023 Indonesia Longitudinal Aging Survey (ILAS). In all, 1982 cross-sectional national data from persons 45 years and older with normal body mass index (18.5–24.9 kg/m^2^) were analysed. Standard measures were used to evaluate anthropometric and independent factors. The factors associated with NWCO were evaluated with Poisson regression. The prevalence of NWCO was 32.8%. Adjusted Poisson regression showed that older age (PR: 1.19, 95% CI: 1.00, 1.45), having diabetes (PR: 1.27, 95% CI: 1.03, 1.57) and hypertension (PR:1.16, 95% CI:1.01, 1.34) were positively associated with NWCO, while being male (PR: 0.23, 95% CI: 0.15, 0.33), being married (PR: 0.73, 95% CI: 0.61, 0.89), working for income (PR: 0.77, 95% CI: 0.63, 0.94), and current smoking (PR: 0.58, 95% CI: 0.36, 0.93) were negatively associated with NWCO. The study showed that about one in three Indonesia’s middle-aged and older persons have NWCO. Findings support the integration of central obesity measures into routine screening to improve early detection of non-communicable diseases (NCDs).

## Introduction

Over 28.5 million Indonesians, or more than 10% of the country’s total population, are 60 years of age or older as of 2020. It is anticipated that by 2050, 20.5% of the population will be 60 years of age or older, which is roughly twice as high as it was in 2020 (Statistics Indonesia, 2023). Indonesia’s population is significantly aging as a result of the epidemiological health transition, and the prevalence of chronic diseases, including obesity, in later life is rapidly rising (Eeuwijk, 2006; Jura & Kozak, 2016). With obesity increasing far more quickly than undernutrition is decreasing, Indonesia’s nutrition shift is speeding up (Muharram et al., 2025). Among middle-aged and older adults in Indonesia, the weighted prevalence of underweight decreased from 10.6% to 26.2%, respectively, in 2007 to 4.6% and 12.4%, respectively, in 2023, the prevalence of obesity (BMI≥ 25 kg/m^2^) increased from 24.5% to 13.1%, respectively, in 2007 to 44.9% and 28.9%, respectively in 2023, and the prevalence of central obesity increased from 28.7% to 24.3%, respectively, in 2007 to 49.2% and 40.6%, respectively in 2023 (Muharram et al., 2025).

The existence of excess abdominal fat in people with a normal body mass index (BMI), known as normal-weight central obesity (NWCO), is becoming more well acknowledged as a serious health issue among older adults (Das et al., 2023). Globally, among adults, the prevalence of NWCO was 21.7% in a multicountry cross-sectional study (Ahmed et al., 2025). It has been generally accepted that general obesity can increase the risk for diabetes, hypertension, and high cholesterol (Blüher, 2019) but also ageing adults with NWCO had a higher prevalence of diabetes (Ahmed et al., 2025), hypertension (Ahmed et al., 2025; Das et al., 2023; Owolabi et al., 2017), high cholesterol (Ahmed et al., 2025), cardiovascular disease, and mortality risk (Coutinho et al., 2013; Sahakyan et al., 2015; Sharma et al., 2016; Xue et al., 2024) than normal-weight individuals. This limitation of BMI is particularly salient in low- and middle-income countries (LMICs) undergoing rapid demographic and epidemiological transitions, such as Indonesia (Popkin et al., 2012; Sudikno et al., 2021), where epidemiological transitions and rising central obesity have been documented using nationally representative data such as the Indonesian Family Life Survey (IFLS) (Megawati et al., 2023).

From a life-course perspective implications of NWCO may differ. Even in the absence of total weight gain, people tend to lose lean muscle mass as they age while gaining visceral adiposity (St-Onge & Gallagher, 2010). BMI often underestimates metabolic risk in middle-aged and older adults due to age-related changes in body composition, such as the loss of muscle mass (sarcopenia) and the increase/redistribution of fat toward visceral areas (Yang et al., 2023). NWCO’s value is further enhanced by placing it within the sociocultural and life-course framework of Indonesia. Indonesia has experienced a fast nutritional transition, marked by a decrease in physical activity and a change from traditional diets high in fiber to energy-dense, processed foods (Popkin et al., 2012). Differential exposure to metabolic risk during the life course is shaped by these changes, which are unevenly distributed between cohorts and geographical areas. For instance, older cohorts may have had undernutrition in their early years followed by overnutrition in their later years, a pattern associated with metabolic vulnerability and central fat deposition (Victora et al., 2008).

NWCO risk is also significantly shaped by gender roles and career paths. Men’s engagement in the workforce in Indonesia has historically involved physically demanding jobs, although this is beginning to change as sedentary employment and urbanization increase (Sander & Yoong, 2020). However, due to caregiving responsibilities, women frequently have irregular work histories, become economically dependent later in life, and have fewer opportunities to engage in physical exercise (Duflo, 2012). Cultural norms around body image and health behaviours may further influence gendered patterns of central obesity, particularly in midlife, when social roles intensify (Abdoli et al., 2024; Maretalinia et al., 2025).

Adopting a cross-cultural and comparative perspective, Indonesia provides a critical case for understanding how NWCO manifests in non-Western settings undergoing rapid social change. Much of the existing literature on NWCO among middle-aged and/or older adults has been derived from high-income countries (Shiraswa et a., 2019), and middle-income countries, such as India (Das et al., 2023) and China (Feng et al., 2021), where ageing trajectories, occupational histories, and family systems may differ to Southeast Asia, such as Indonesia (Kapoor et al., 2019). Factors linked to NWCO among adults may include female sex (Feng et al., 2021; Guerra Valencia et al., 2023; Mohamed et al., 2019; Zhang et al., 2016), older age (Feng et al., 2021; Guerra Valencia et al., 2023; Mohamed et al., 2019; Zhang et al., 2016), higher wealth status (Guerra Valencia et al., 2023), full-time employment (Mohamed et al., 2019), retired, unemployed or others (Zhang et al., 2016), hypertension (Feng et al., 2021; Guerra Valencia et al., 2023), diabetes (Guerra Valencia et al., 2023), dislipidemia (Feng et al., 2021), non-daily cigarette smoking (Feng et al., 2021).

By examining NWCO among middle-aged and older adults in Indonesia, insights may have broader implications for other LMICs experiencing similar demographic ageing and nutrition transitions. Understanding NWCO in this context can inform cross-national comparisons and guide the development of more nuanced, age-sensitive, and culturally appropriate health assessments and interventions. The aim of this study was to investigate the prevalence and related factors of NWCO among people aged 45 and over, using national community-dwelling data from the 2023 Indonesia Longitudinal Aging Survey (ILAS). By integrating gerontological and cross-cultural perspectives, we aim to advance understanding of how central obesity within the normal BMI range reflects broader processes of ageing, social change, and health inequality.

## Methods

### Study Design and Procedures

The current analyses utilized data from the 2023 Indonesia Longitudinal Aging Survey (ILAS) (Asian Development Bank, etc., 2024). Nine provinces in all were chosen to serve as the study areas. When choosing a sample, multistage random sampling is employed, accounting for socioeconomic factors, urban-rural disparities, and the proportion of the population that is 45–69 and 70 years of age or older. Respondents are chosen at random from 17 houses in each chosen village that have at least one person 45 years of age. The response rate was 97.8% (Asian Development Bank, etc., 2024). The sample with complete anthropometric responses was 3865.

### Ethical Statement

With reference number 558/KE.01/SK/12/2022, the Ethical Commission for Social and Humanity and the National Research and Innovation Agency (Badan Riset dan Inovasi Nasional) granted ethical approval, and participants provided written informed consent (Asian Development Bank, etc., 2024).

### Measures

### Dependent and Exposure Variables

During the study, trained field workers used conventional protocols to take anthropometric measurements, such as height, weight, and waist circumference. The Omron digital scale was used to measure weight in kilograms (kg). A stadiometer with a 2 m capacity and 0.1 cm precision was used to measure height. To the closest 0.1 cm, the waist circumference was measured at the halfway point between the superior border of the iliac crest and the lower border of the rib cage.

Body mass index (BMI) was calculated as weight in kilograms divided by height in meters squared (kg/m^2^). Normal general weight was classified as a BMI of 18.5–24.9 kg/m^2^.

(Sruthi et al., 2025). Central obesity was classified as individuals who had waist circumference ≥ 80 cm for women and ≥ 90 cm for men (based on the Asian population) (Alberti et al., 2005). NWCO was defiend as having a normal BMI in combination with central obesity (Das et al., 2023; Peltzer & Pengpid, 2026). Study participants who were overweight/obese (BMI ≥ 25 kg/m^2^) or underweight (BMI < 18.5 kg/m^2^) were excluded from this analysis.

Sociodemographic factors included age, sex (male, female), work for income (Yes/No), and religion (Islam versus Protestant/Catholic/Hindu/Buddhism/Confucianism/Indigenous faith/other), marital status (single/never married, separated, divorced, widow/er = 1 and married, cohabitate = 0), education (≥ secondary = 1 and < secondary = 0), personal income, residence status (rural-urban), and internet use. *Personal income* was assessed with the question, “What type of income or payment did you receive in the last 12 months?” including (1) Pension, (2) Rent, (3) Salary/Income from business, (4) Insurance, (5) Dividends from shares/deposit interest, (6) Abandoned Elderly Social Assistance Program (ASLUT), (7) BPNT (basic foods) for the Elderly/individual, Family Hope Program (PKH) for the Elderly/individual, (8) Assistance for the elderly/individual from the Regency/City, (9) *Bantuan Langsung Tunai* (BLT) scheme for elderly/individual, and (10) Other. Summed personal income was sub-divided into low (< 6 million IDR), and high (≥ 6 million IDR). In 2023, 1 million Indonesian rupiah (IDR) was worth approximately $65.60 to $69.08 USD. *Internet use* was assessed with the item, “How often do you access e-mail, the internet, social media, watch videos, or other purposes using a computer/smartphone?” Rarely-Always = 1 and Never = 0) (Asian Development Bank, etc., 2024).

### Physical and Mental Health Risk Status

### Poor Self-Rated Health Status

“Which of the following best describes your current health status?” Responses ranged from 1 = very good to 5 = very poor

Diabetes and high cholesterol is reported by ever having been diagnosed by a doctor or health worker.

### Hypertension

A digital blood pressure monitor (OMRON) is used to take the blood pressure readings for this survey. The average of three successive measurements of systolic and diastolic blood pressure (BP) were recorded, and ‘Hypertension was defined as systolic BP ≥ 140 mm Hg and/orDBP ≥ 90 mm Hg.’ (Chobanian et al., 2003).

Number of pain sites. “Did you experience any pain in the […] body parts in the last 30 days that limit/disrupt your daily activities, now?” including “(1) head (yes/no), (2) neck, (3) shoulder, (4) arms, (5) wrist, (6) fingers, (7) chest, (8) stomach, (9) back, (10) hips, (11) legs, (12) knees, (13) ankles, and (14) toes” (yes/no). Responses of all 14 pain sites were summed. Cronbach’s alpha was 0.89 in this study.

Depressive symptoms were measured using the ‘Centers for Epidemiologic Studies Depression Scale’ (CES-D-10). Ten questions about respondents’ emotions throughout the previous week are part of the CES-D 10 module. If a respondent’s total score is 10 or more, they are classified with having depressive symptoms (Andresen et al., 1994). Cronbach’s alpha was 0.81 in this study. The CES-D-10 has been validated as a reliable and valid instrument for older adults in Indonesia (Priyadi, 2023).

## Health Risk Behaviour

Physical Activity. ”How Often Do You Go for a Walk/Jog/Gym?” Response Options for These Items Ranged From 1 = Never to 5 = Always

Current smoking. “Do you currently smoke? Including smoke shisha, cigarettes, cigars, pipe with tobacco, chewing tobacco, etc.?” (Yes/No).

History of alcohol use. “Do you have the custom of consuming alcoholic beverages such as beer, wine, arrack or toddy, and others?” (Yes/No) (Asian Development Bank, etc., 2024).

### Statistical Analysis

The sample and the prevalence of the NWCO were described using descriptive statistics. Chi-square and ANOVA tests were used to calculated differences in proportions. Poisson regression was applied to estimate prevalence ratios (PR) and 95% Confidence Interval (CI) of NWCO. Variables that were found significant (*p* < 0.05) in univariable analysis (age group, sex, marital status, work for income, personal income, lower self-rated health, diabetes, hypertension, high cholesterol, number of pain sites, current smoking and history of alcohol use) were included in the multivarible model. Independent variables were introduced based on a previous literature analysis (Feng et al., 2021; Guerra Valencia et al., 2023; Mohamed et al., 2019; Zhang et al., 2016). To take into consideration the complex sampling design, weights were applied to both regression analyses and descriptive statistics. Based on the percentage of the chosen samples in the population, each enumeration area is given a weight. The hierarchy or level is taken into account while calculating the weights, starting with the districts and municipalities and concluding with the selected villages. The gender variable is used as a control when calculating individual weights (Asian Development Bank, etc., 2024). There was no evidence of collinearity in the Variance Inflation Factor (VIF). All tests were considered statistically significant at *p* < 0.05. The STATA programme (Stata Corporation, College Station, Texas, USA) version 18.0 was used for all statistical procedures.

## Results

### Sample Characteristics

In total, 1982 participants (mean age 58.9 years, SD = 9.4 years, range 45–102 years) with normal body weight (BMI 18.5–24.9 kg/m^2^) were included in the sample. The prevalence of NWCO was 32.8%, which differed by age, sex, marital status, personal income, work status, internet use, having diabetes, hypertension, high cholesterol, number of pain sites, current smoking and history of alcohol use (see Table 1).

**Table 1:** Characteristics of the sample and normal weight central obesity, 45 years and older, ILAS 2023

Variable	Sample	Normal weight central obesity		P-value^a^
		No	Yes	
	N (%)	N (%)/M (SD)	N (%)/M (SD)	
All	1982	1300 (67.2)	682 (32.8)	
Sociodemographics				
Age in years: N (%)				
45-59	1090 (58.8)	767 (71.7)	323 (28.3)	<0.001
60-102	892 (41.2)	533 (60.9)	359 (39.1)	
Sex: N (%)				
Female	949 (45.9)	375 (40.0)	574 (60.0)	<0.001
Male	1033 (54.1)	925 (90.3)	108 (9.7)	
Education: N (%)				
<Secondary	1204 (65.0)	778 (67.1)	426 (32.9)	0.257
≥Secondary	778 (35.0)	522 (67.5)	256 (32.5)	
Marital status: N (%)				
Not married	503 (25.5)	250 (50.2)	253 (49.8)	<0.001
Married	1479 (74.5)	1050 (73.1)	429 (26.9)	
Religion: N (%)				
Other	230 (6.5)	150 (59.0)	80 (41.0)	0.899
Islam	1752 (93.5)	1150 (67.8)	602 (32.2)	
Personal income: N (%)				
Low	929 (45.4)	537 (58.0)	392 (42.0)	<0.001
High	1053 (54.6)	763 (75.0)	290 (25.0)	
Work for income: N (%)	1473 (73.9)	1060 (74.1)	413 (25.9)	<0.001
Internet use: N (%)	615 (32.3)	431 (71.7)	184 (28.3)	0.005
Residence status: N (%)				
Rural	838 (37.4)	569 (67.6)	269 (32.4)	0.064
Urban	1144 (62.6)	731 (67.0)	413 (33.0)	
Physical and mental health risk status				
Lower self-rated health (1-5): M (SD)	2.57 (0.7)	2.56 (0.7)	2.60 (0.7)	0.251
Diabetes: N (%)	127 (6.4)	50 (42.2)	77 (57.8)	<0.001
Hypertension: N (%)	969 (52.6)	596 (63.3)	373 (36.7)	<0.001
High cholesterol: N (%)	270 (13.6)	146 (54.9)	124 (45.1)	<0.001
Number of pain sites (0-14): M (SD)	2.70 (3.3)	2.55 (3.2)	2.98 (3.5)	0.006
Depressive symptoms: N (%)	140 (7.8)	95 (69.5)	45 (30.5)	0.424
Health risk behaviour				
Physical activity (1-5): M (SD)	2.29 (1.5)	2.25 (1.4)	2.38 (1.5)	0.058
Current smoking: N (%)	638 (35.2)	573 (91.6)	65 (8.4)	<0.001
History of alcohol use: N (%)	122 (7.3)	104 (90.6)	18 (9.4)	<0.001

### Associations with NWCO

Adjusted Poisson regression showed that older age (PR: 1.19, 95% CI: 1.00, 1.45), having diabetes (PR: 1.27, 95% CI: 1.03, 1.57) and hypertension (PR:1.16, 95% CI:1.01, 1.34) were positively associated with NWCO, while being male (PR: 0.23, 95% CI: 0.15, 0.33), being married (PR: 0.73, 95% CI: 0.61, 0.89), working for income (PR: 0.77, 95% CI: 0.63, 0.94), and current smoking (PR: 0.58, 95% CI: 0.36, 0.93) were negatively associated with NWCO (see Table 2).

**Table 2:** Associations with normal weight central obesity, 45 years and older, ILAS 2023

Variable	CPR (95% CI)	APR (95% CI) ^a^
Sociodemographics		
Age in years		
45–59	1 (Reference)	1 (Reference)
60–102	1.38 (1.16, 1.64)***	1.19 (1.00, 1.45)*
Sex		
Female	1 (Reference)	1 (Reference)
Male	0.16 (0.12, 0.22)***	0.23 (0.15, 0.33)***
Education (≥ Secondary)	0.99 (0.81, 1.21)	---
Marital status (Married)	0.54 (0.45, 0.64)***	0.77 (0.61, 0.89)***
Work for income	0.49 (0.42, 0.59)***	0.77 (0.63, 0.94)**
Personal income (high)	0.60 (0.50, 0.72)***	0.89 (0.73, 1.08)
Internet use	0.81 (0.65, 1.01)+	---
Religion		
Other	1 (Reference)	---
Islam	0.78 (0.60, 1.03)+	
Residence status		
Rural	1 (Reference)	---
Urban	1.02 (0.85, 1.21)	
Physical and mental health risk status		
Lower self-rated health	1.14 (1.01, 1.28)*	1.07 (0.95, 1.22)
Diabetes	1.86 (1.45, 2.39)***	1.27 (1.03, 1.57)*
Hypertension	1.28 (1.07, 1.53)**	1.16 (1.01, 1.34)*
High cholesterol	1.46 (1.17, 1.83)***	1.05 (0.85, 1.30)
Number of pain sites	1.04 (1.01, 1.06)**	1.00 (0.98, 1.03)+
Depressive symptoms	0.94 (0.66, 1.35)	---
Health risk behaviour		
Physical activity	1.04 (0.98, 1.10)	---
Current smoking	0.18 (0.12, 0.27)***	0.58 (0.36, 0.93)*
History of alcohol use	0.27 (0.14, 0.53)***	0.94 (0.46, 1.91)

^a^Adjusted for all variables in the table, *CPR* Crude Prevalence Ratio, *APR* Adjusted Prevalence Ratio, *CI* Confidence Interval; +*p* < 0.1, *p < 0.05, **p < 0.01, ***p < 0.001

## Discussion

This study found that nearly one in three Indonesian adults aged ≥ 45 years with normal BMI had normal-weight central obesity (NWCO), underscoring a substantial hidden cardiometabolic risk burden that would be missed by BMI-based screening alone. The found prevalence of 32.8% NWCO among people with normal BMI is consistent with evidence from adults in LMICs, e.g. 34.0% in Mongolia (Peltzer & Pengpid, 2026).

In line with gerontological data that ageing is accompanied by sarcopenia, fat redistribution toward the belly, and hormonal changes that encourage visceral fat storage, older age was positively correlated with NWCO (Zamboni et al., 2008). This supports the idea of sarcopenic obesity and suggests that as people age, their BMI becomes less responsive to the danger associated with obesity. The metabolic significance of central adiposity, which is closely associated with insulin resistance and cardiometabolic diseases, is reinforced by the positive correlation between diabetes and NWCO (Després, 2012). The increased risk of developing metabolic disorders may account for the correlations between hypertension and NWCO (Feng et al., 2021).

In line with sex-specific fat distribution patterns and the impact of menopause on central fat accumulation, men had a much lower prevalence of NWCO than women (Das et al., 2023; Lovejoy et al., 2008; Prasad et al., 2020). Additionally, social variables were found to be significant: lower NWCO frequency was linked to marriage and income-generating employment, which may indicate more social support, increased physical activity, and regimented daily schedules (Guerra Valencia et al., 2023; Umberson & Montez, 2010). Although smoking is occasionally connected to decreased body weight, it is also associated with greater central fat deposition and serious health hazards, therefore the inverse association with current smoking should be read cautiously (Chiolero et al., 2008).

Overall, these findings highlight the limitations of BMI as a standalone indicator in ageing populations and emphasize the importance of incorporating measures of central obesity (e.g., waist circumference) into routine health assessments (WHO, 2011). Given that BMI alone may lead to incorrect classifications and underestimating of at-risk persons, the high prevalence of NWCO discovered in this study highlights the necessity of using anthropometric indicators in addition to BMI when measuring excessive bodyweight. Given this, it’s critical to use a mix of waist circumference and BMI when screening for NWCO. In order to treat and/or prevent the development of cardio-metabolic issues, such persons would typically need to be provided with the proper health education and quick intervention. Additionally, it offers a fresh viewpoint on how to prevent metabolic illnesses by monitoring your waist circumference, even if your BMI is normal (Feng et al., 2021).

### Study Strengths and Limitations

One of the study’s strengths was the use of standardized measures that were adapted from the Health and Retirement Study in conjunction with a national sample of middle-aged and older persons in Indonesia. However, the study included only community-dwelling individuals, potentially excluding frailer or institutionalized older adults Most statistics may have limitations due to self-reporting, such as diabetes and high cholesterol. Furthermore, because the study’s data were cross-sectional, we are unable to prove a causative association between any of the associated characteristics. To address this limitation future longitudinal studies are needed. Important variables, such as dietary behaviour and menopausal status (Syauqy et al., 2025), that could be associated with body weight status were not assessed and should be included in future studies.

## Conclusions

NWCO is highly prevalent among middle-aged and older adults with normal BMI in Indonesia and is associated with older age, diabetes and hypertension, while being less common among men, married individuals, those working, and current smokers. These findings reveal a significant hidden burden of cardiometabolic risk not captured by BMI alone and support the integration of central obesity measures into routine screening to improve early detection of non-communicable diseases (NCDs).

## Implications for Health Systems and Ageing Policy

Primary care services should include waist circumference alongside BMI to improve identification of high-risk individuals (WHO, 2011). Clinical and public health guidelines.

and ageing strategies should recognize NWCO as a distinct risk category requiring targeted prevention and management. Target high-risk subgroups of NWCO, such as older adults, women, and individuals with diabetes and hypertension for interventions focusing on abdominal obesity reduction. As part of integrated NCD management, NWCO screening should be incorporated into diabetes and hypertension programmes to enhance early detection and management (Després, 2012). Policies encouraging continued employment and social engagement may indirectly support healthier body composition (Umberson & Montez, 2010).

Healthcare workers should be trained to measure and interpret central obesity indicators and communicate associated risks effectively.

## Data Availability

SurveyMETER, https://www.surveymeter.org/ilas/.
